# Early anal gland adenocarcinoma with a characteristic submucosal tumor-like appearance: a case report

**DOI:** 10.1186/s12957-018-1451-1

**Published:** 2018-07-21

**Authors:** Mikito Mori, Keiji Koda, Atsushi Hirano, Kiyohiko Shuto, Chihiro Kosugi, Kazuo Narushima, Isamu Hosokawa, Takayuki Suzuki, Masato Yamazaki, Hiroaki Shimizu

**Affiliations:** Department of Surgery, Teikyo University Chiba Medical Center, 3426-3 Anesaki, Ichihara, Chiba 299-0111 Japan

**Keywords:** Case report, Anal cancer, Early anal canal cancer, Anal gland carcinoma, Anal mucinous adenocarcinoma

## Abstract

**Background:**

The clinical findings of early anal gland carcinoma (AGC) have not been well delineated because AGC is a rare malignancy usually diagnosed at an advanced stage. Knowledge of the characteristic findings will be helpful for both diagnosis and determination of the treatment options for early AGC.

**Case presentation:**

A 62-year-old man was referred to our hospital for treatment of a rectal submucosal tumor (SMT) detected during a medical checkup at another hospital. Trans-sacral resection of the tumor was performed under the diagnosis of a rectal benign cyst. Pathological examination of the resected tumor showed a mucin-producing adenoma. About 14 months later, a new cystic lesion was found by follow-up examination, and trans-sacral resection of the tumor was performed again. The second pathological diagnosis was a mucinous adenocarcinoma with a possible remnant tumor at the local site. After providing sufficient informed consent, the patient underwent intersphincteric resection (ISR) of the rectum to preserve anal function. The final diagnosis was mucinous adenocarcinoma of the anal gland, T1N0M0. The patient remained alive without recurrence or complications for 6 years 7 months postoperatively.

**Conclusion:**

We have herein reported a case of early AGC with a characteristic SMT-like appearance. Because the anal gland is located within both the submucosal layer and the internal sphincter muscle, ISR may be selected when the tumor is limited to inside the gland.

## Background

Adenocarcinomas of the anal gland are rare malignancies that are found in an advanced stage in most patients [1]. Mucinous adenocarcinoma of the anal canal is also a rare malignancy. It is often associated with an anal fistula and is also generally found in an advanced stage [2]. We herein report a rare case of an early anal gland mucinous adenocarcinoma that showed a characteristic submucosal tumor (SMT)-like appearance and was successfully treated with an anal sphincter-preserving operation.

## Case presentation

**Fig. 1:**
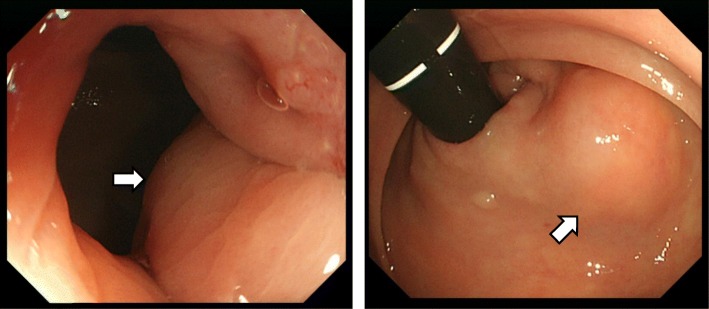
Lower gastrointestinal endoscopy findings. A submucosal tumor-like region was seen on the right anterior wall of the rectum. The tumor had no mucosal defects, and its surface was normal

**Fig. 2:**
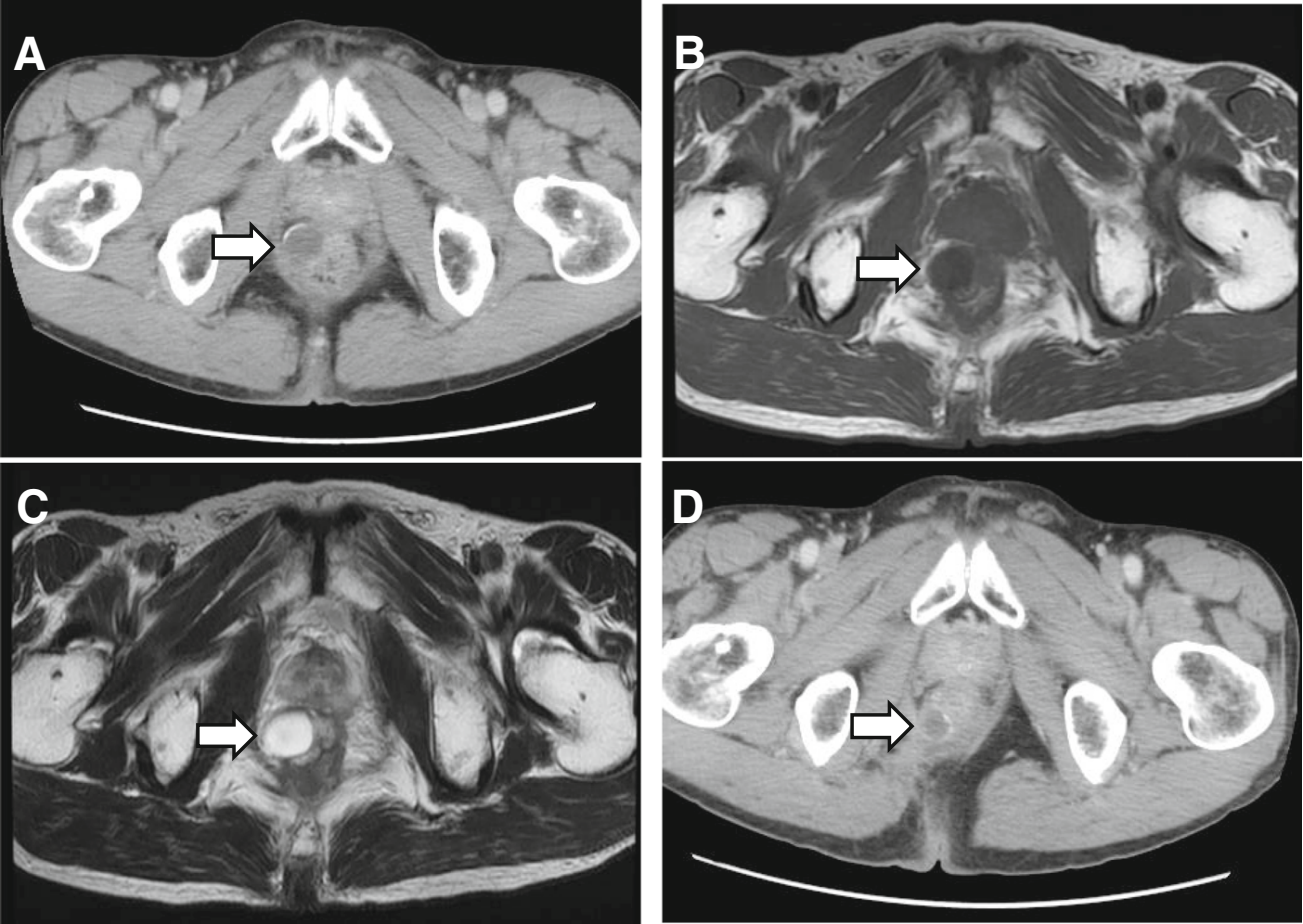
Abdominal (**a**) computed tomography images, **b** T1-weighted magnetic resonance images, and (**c**) T2-weighed magnetic resonance images prior to the first operation. A submucosal cystic lesion without enhancement of the capsule was seen on the right side of the lower rectal wall. It contained homogeneous fluid with calcification of the capsule. **d** A similar tumor was detected by computed tomography 14 months after the first operation

**Fig. 3:**
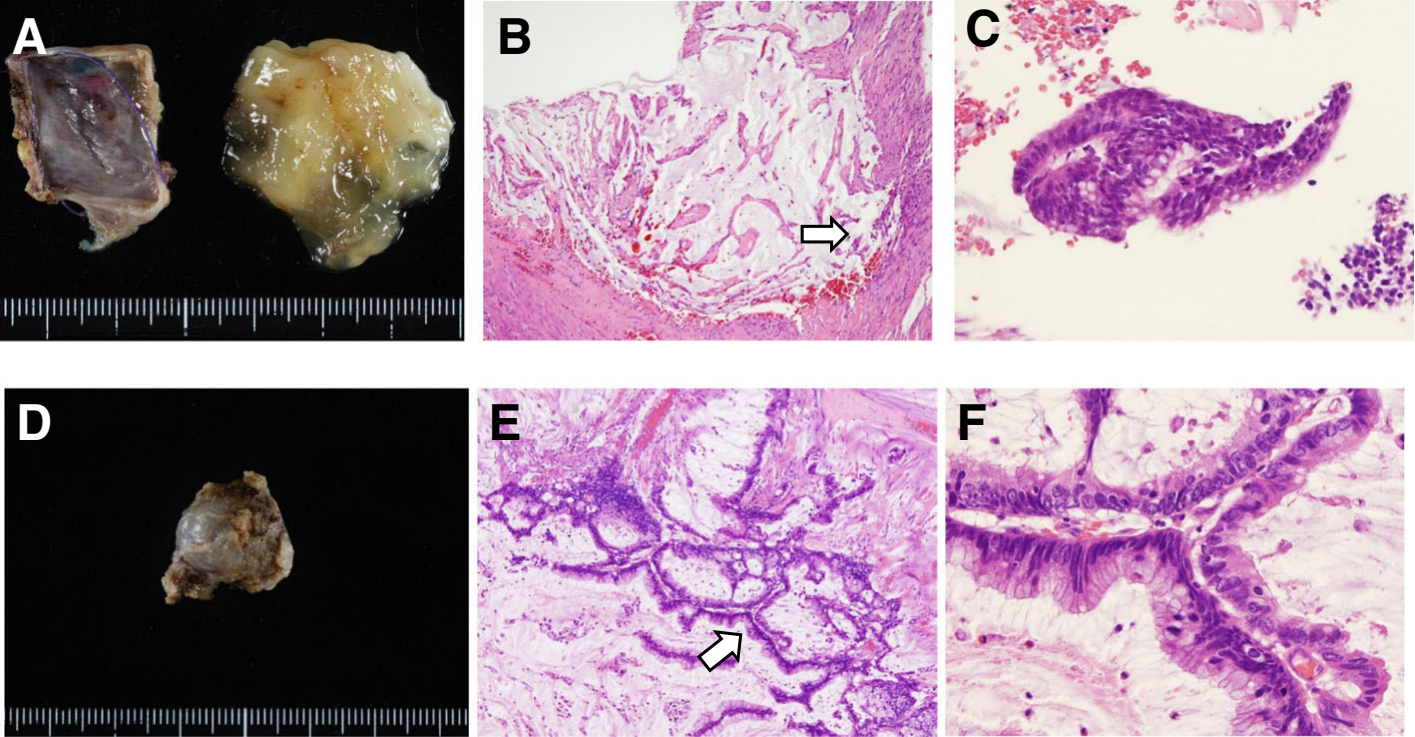
Histopathological findings of the resected specimen at (**a–c**) the first operation and (**d–f**) the second operation. **a** Retention of yellow jelly-like mucus in the cavity. **b**, **c** High-grade adenoma with mucin production. **d** The capsule containing the mucus. **e**, **f** Mucinous adenocarcinoma limited to the anal gland [hematoxylin and eosin staining at (**a**, **d**) × 20, (B, E) × 40, and (**c**, **f**) × 100. Arrows in B and E indicate areas shown in C, F, respectively]

**Fig. 4:**
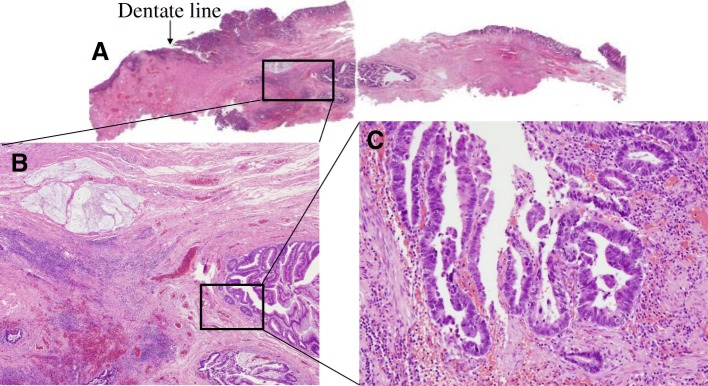
Histopathological findings of intersphincteric resection with partial external sphincter resection [hematoxylin and eosin staining at (**a**) × 20, (**b**) × 40, and (**c**) × 100 magnification]. The tumor was limited to the anal gland and did not invade deeper areas

**Fig. 5:**
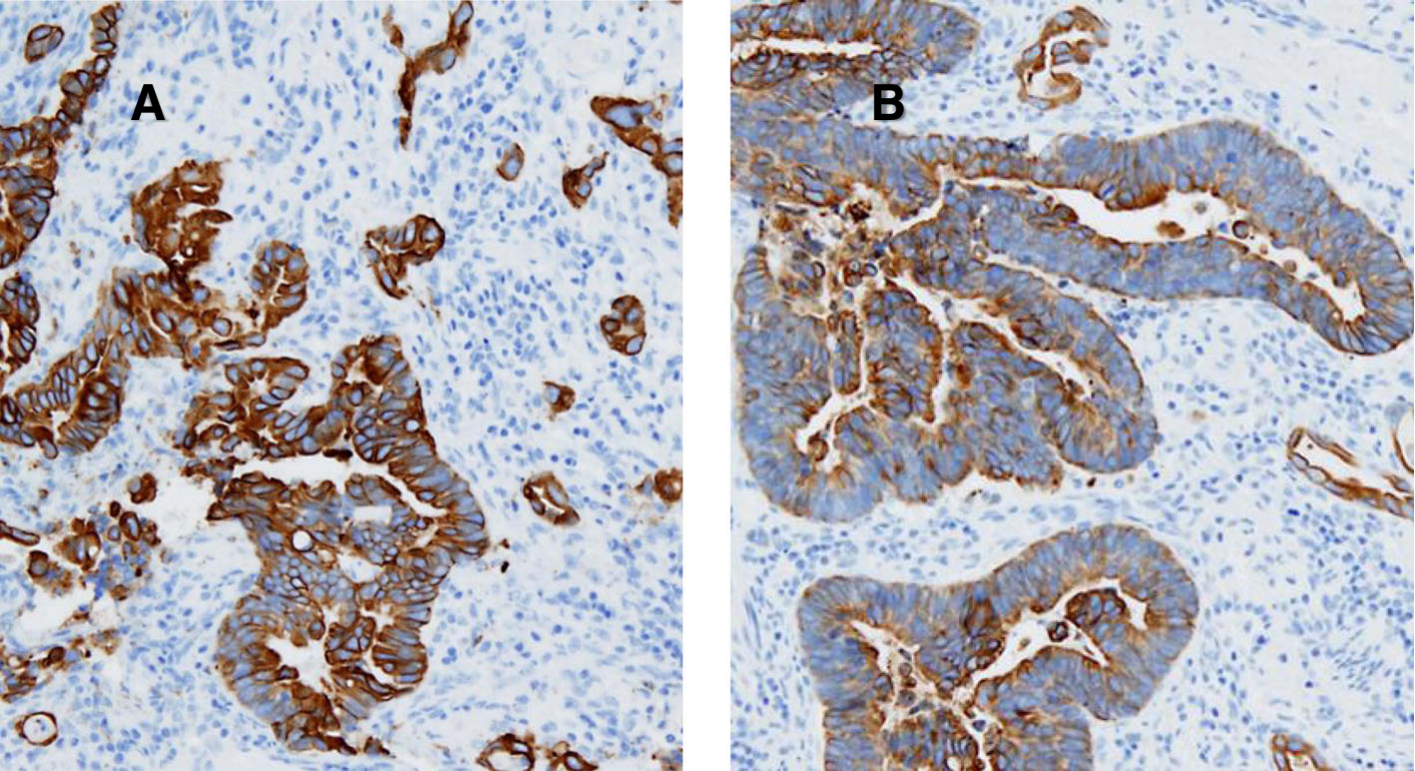
Immunohistochemical analysis showing that the tumor cells were (**a**) positive for cytokeratin 7 (× 100 magnification) and (**b**) negative for cytokeratin 20 (× 100 magnification)

A 62-year-old man was referred to our hospital for treatment of a rectal SMT detected during a medical checkup at another hospital. Digital examination of the anus and rectum revealed a 20-mm elastic hard tumor palpable on the right and ventral sides of the anal canal and located 2 to 3 cm proximal to the anal verge. Laboratory examination showed no elevation of either carcinoembryonic antigen or cancer antigen 19-9. Colonoscopy showed a 20-mm SMT in the anal canal (Fig. 1). Abdominal computed tomography (CT) showed a 20-mm cystic tumor on the right site of the lower rectum with no evidence of lymph node or distant metastases (Fig. 2a). Magnetic resonance imaging (MRI) also showed a 20-mm cystic tumor on the right site of the lower rectum (Fig. 2b, c). These findings strongly suggested a benign cyst in the anal canal; therefore, the patient underwent trans-sacral resection for precise diagnosis of the tumor. The pathological diagnosis of the resected tumor was a mucinous adenoma with high-grade dysplasia (Fig. 3a–c) with negative surgical margins. The patient was observed in ambulatory practice. Follow-up CT and MRI 14 months after surgery showed a new cystic lesion near the site of the removed tumor (Fig. 2d). To evaluate whether the new cystic tumor was a recurrence, the patient underwent trans-sacral resection of the cystic tumor again. Pathological examination of the second resected tumor revealed that the tumor was a mucinous adenocarcinoma of the lower rectum (Fig. 3d–f) with a possible remnant tumor at the local site. After providing sufficient informed consent, the patient underwent anal sphincter-preserving intersphincteric resection (ISR) with partial resection of the external sphincter along with prophylactic lymph node dissection. Pathological examination showed that the tumor cells were located at the anal gland under the mucosa of the anal canal and that these cells produced mucin and fibrosis (Fig. 4). Immunohistochemical analysis showed that the tumor cells were positive for cytokeratin 7 (CK7) and negative for CK20 (Fig. 5). The final diagnosis was an early mucinous adenocarcinoma arising from the anal gland without an invasive appearance. The TNM classification was pT1N0M0 stage I, and the surgical margin was negative for tumor tissue. No adjuvant chemotherapy was performed for this patient. At the time of this writing, 79 months after surgery, the patient was alive without recurrence and active in society.

## Discussion and conclusions

Carcinomas of the anal canal are rare malignancies, comprising approximately 1 to 2% of all gastrointestinal carcinomas [3]. Most anal canal carcinomas are squamous (epidermoid) cell carcinomas [4], and mucinous adenocarcinoma accounts for approximately 3% of all anal canal cancers [5]. Adenocarcinoma arising from the columnar epithelium of the upper anal canal resembles rectal adenocarcinoma, showing CK7 negativity and CK20 positivity on immunohistochemical staining. Adenocarcinomas also arise in anorectal fistulas or in the anal glands. In particular, approximately 80% of anal mucinous adenocarcinomas are reportedly associated with anorectal fistulas [2]. Anal gland carcinomas (AGCs) are very rare [6, 7]. Such tumors show positive immunohistostaining for CK7 and negative staining for CK20 [8–11]. In many cases, affected patients have symptoms such as anal pain, rectal bleeding, discharge, or a perianal mass [12], all of which are associated with advanced-stage diagnoses [2]. In addition, approximately half of AGCs are reportedly associated with anal fistulas [1]. In our case, the patient had no symptoms but had been previously diagnosed with an SMT-like rectal tumor during a regular health check-up. The tumor was SMT-like in shape, located at the lower rectum on colonoscopic examination, and cyst-like in appearance on CT and MRI. Because the opening of the anal gland is located in the transitional zone at the level of the dentate line and the gland extends into the submucosa and internal sphincter [13], an early mucinous adenocarcinoma that is limited to within the anal gland may exhibit an SMT- and cyst-like appearance, as seen in our patient. The characteristic SMT-like appearance and cystic features on CT or MRI provide important information in the potential diagnosis of early stage anal gland carcinoma. An immunohistochemical study using either fine needle biopsy or local excision of the tumor is necessary for the precise diagnosis.

Although local excision can be performed in some cases when the tumor is limited in its location [1, 12], the local excision we performed in the first operation may have resulted in tumor residue in the other lobes of the anal gland. Therefore, after local removal of the remnant cystic tumor during the second operation and obtaining a pathological diagnosis of mucinous adenocarcinoma, we performed ISR with temporary diverting ileostomy for total removal of the anal gland. In many cases, abdominoperineal resection may be selected as an additional operation because AGCs are often diagnosed in an advanced stage. However, if the carcinoma is in an early stage and is limited to within the anal gland, an anus-preserving operation may be considered by total removal of the anal gland using an ISR technique [14]. The patient in the present case was still alive without recurrence and active in society at the time of this writing (79 months after the final operation) despite experiencing some defecation-related difficulty inherent to ISR. Close postoperative follow-up including local digital examination, blood tests, and CT is necessary for early detection of both local and distant recurrence, especially when an anal sphincter-preserving operation is selected.

In conclusion, we have described a case of early stage mucinous adenocarcinoma arising from the anal gland with a characteristic SMT-like cystic appearance. When the tumor spread is limited to within the anal gland, an anal sphincter-preserving operation may be considered in addition to abdominoperineal resection for total removal of the anal gland even after local excision has failed.

## Abbreviations

AGC: Anal gland carcinoma
CT: Computed tomography
ISR: Intersphincteric resection
MRI: Magnetic resonance imaging
SMT: Submucosal tumor
